# Context and Expectations Matter: Social, Recreational, and Independent Functioning among Youth with Psychosis in Chennai, India and Montreal, Canada

**DOI:** 10.1177/07067437231153796

**Published:** 2023-02-06

**Authors:** Srividya N. Iyer, Thara Rangaswamy, Sally Mustafa, Nicole Pawliuk, Greeshma Mohan, Ridha Joober, Norbert Schmitz, Howard Margolese, Ramachandran Padmavati, Ashok Malla

**Affiliations:** 1 Department of Psychiatry, 5620 McGill University, Montreal, Canada; 2 Prevention and Early Intervention Program for Psychosis (PEPP-Montreal), 26632 Douglas Mental Health University Institute, Montreal, Canada; 3 29868 Schizophrenia Research Foundation (SCARF), Chennai, India; 4 Department of Population-Based Medicine, Institute of Health Sciences, University Hospital Tübingen, Tübingen, Germany

**Keywords:** first-episode psychosis, early intervention, cross-cultural, functioning, recreation, leisure, social, LMICs

## Abstract

**Objectives:**

Most cross-cultural psychosis research has focused on a limited number of outcomes (generally symptom-related) and perspectives (often clinician-/observer-rated). It is unknown if the purported superior outcomes for psychosis in some low- and middle-income countries extend to patient-reported measures of social, recreational, and independent functioning. Addressing this gap, this study aimed to compare these outcomes in first-episode psychosis at a high-income site and a lower middle-income site.

**Methods:**

Patients receiving similarly designed early intervention for psychosis in Chennai, India (*N* = 164) and Montreal, Canada (*N* = 140) completed the self-reported Social Functioning Scale-Early Intervention, which measures prosocial, recreation, and independence-performance functioning. Their case managers rated expected independence-performance functioning. Both sets of assessments were done at entry and Months 6, 18, and 24. Linear mixed model analyses of differences between sites and over time were conducted, accounting for other pertinent variables, especially negative symptoms.

**Results:**

Linear mixed models showed that prosocial, recreation, and independence-performance functioning scores were significantly higher in Montreal than Chennai and did not change over time. Expected independence-performance was also higher in Montreal and increased over time. Negative symptoms and education independently predicted prosocial, recreation, and expected independence-performance functioning. When added to the model, expected independence-performance predicted actual independence-performance and site was no longer significant. At both sites, prosocial and recreation scores were consistently lower (<40%) than independence-performance (40–65%).

**Conclusion:**

This is the first cross-cultural investigation of prosocial, recreation, and independent functioning in early psychosis. It demonstrates that these outcomes differ by socio-cultural context. Differing levels of expectations about patients, themselves shaped by cultural, illness, and social determinants, may contribute to cross-cultural variations in functional outcomes. At both sites, social, recreational, and independent functioning were in the low-to-moderate range and there was no improvement over time, underscoring the need for effective interventions specifically designed to impact these outcomes.

## Résumé

**Objectifs:**

La majorité de la recherche sur la psychose interculturelle a porté sur un nombre limité de résultats (généralement liés aux symptômes) et de perspectives (souvent évalués par un clinicien/observateur). Il demeure inconnu si les résultats prétendument supérieurs de la psychose dans des pays à revenu faible et moyen s’étendent à des mesures déclarées par le patient du fonctionnement social, récréatif et indépendant. Pour combler cet écart, la présente étude visait à comparer ces résultats au premier épisode de psychose à un endroit à revenu élevé et à revenu moyen inférieur.

**Méthodes:**

Les patients recevant des interventions précoces de même conception pour la psychose à Chennai, Inde (*N* = 164) et Montréal, Canada (*N* = 140) ont rempli l’échelle de fonctionnement social-intervention précoce (auto-déclarée), qui mesure le fonctionnement pro-social, récréatif et d’indépendance- performance. Leurs gestionnaires de cas ont évalué le fonctionnement prévu d’indépendance- performance. Les deux ensembles d’évaluations ont été faits à l’entrée et aux 6^e^, 18^e^ et 24^e^ mois. Les analyses de modèle linéaire mixte des différences entre les sites et avec le temps ont été menées, en prenant compte d’autres variables pertinentes, spécialement les symptômes négatifs.

**Résultats:**

Les modèles linéaires mixtes ont montré que les scores de fonctionnement pro-social, récréatif et d’indépendance-performance étaient significativement plus élevés à Montréal qu’à Chennai et ne changeaient pas avec le temps. Le rendement indépendant prévu était également plus élevé à Montréal et augmentait avec le temps. Les symptômes négatifs et l’éducation prédisaient indépendamment le fonctionnement pro-social, récréatif et d’indépendance-performance prévu. Lorsqu’ajoutée au modèle, l’indépendance-performance prévue prédisait la performance indépendante réelle et le site n’était plus significatif. Aux deux sites, les scores au fonctionnement pro-social et récréatif étaient régulièrement plus faibles (< 40%) que l’indépendance-performance (40–65%).

**Conclusion:**

C’est la première recherche interculturelle du fonctionnement pro-social, récréatif et indépendant dans la psychose précoce. Elle démontre que ces résultats diffèrent selon le contexte socioculturel. Des niveaux différents d’attentes à propos des patients, eux-mêmes formés par des déterminants culturels, sociaux et de la maladie, peuvent contribuer à des variations interculturelles dans les résultats fonctionnels. Dans les deux sites, les fonctionnements social, récréatif et indépendant étaient dans la gamme faible à modérée et il n’y avait pas d’amélioration avec le temps, ce qui souligne le besoin d’interventions efficaces spécifiquement conçues pour influencer les résultats.

## Introduction

Most of the evidence showing that early intervention services for psychosis improve outcomes, like symptom severity, treatment discontinuation, hospitalization, and functional roles, comes from high-income countries (HICs).^1^ Early intervention services have rarely been focused on in cross-cultural psychosis research, partly due to the paucity of early psychosis services in low- and middle-income countries (LMICs).^2^ Cross-cultural psychosis research has also been critiqued for focusing on select outcomes (typically symptom-related) and perspectives (often clinician- or observer-rated).^3^ We have therefore been comparing multiple outcomes (chosen a priori) among persons with first-episode psychosis receiving similar early intervention in an HIC (Montreal, Canada) and an LMIC (Chennai, India) context.^4–6^ We also broadened the scope of outcomes and perspectives by assessing cross-cultural differences in patient-reported social, recreational, and independent functioning. These aspects of functional outcomes have been recognized as crucial to recovery and well-being.^7–9^ Functioning in these domains can also help in coping with or reducing psychiatric symptoms.^10,11^

Normative expectations about functioning are shaped by geo-sociocultural context and associated value orientations. Differences in value orientations across countries have been the subject of much research.^12^ While such work has been criticized for minimizing diversity within countries,^13^ value orientations can help unpack contextual differences in studied phenomena. Schwartz's embeddedness versus autonomy value orientation is especially salient in this study.^12^ In the Indian context, there is a greater emphasis on embeddedness, that is, familial, group, and religious affiliations and obligations, whereas in Canada, there is a greater emphasis on intellectual and affective (via pleasurable/exciting/varied experiences) autonomy.^5,14–18^ There are also sociocultural differences in expectations around adulthood. In India, the coming of age is often significantly later than the technical age of majority and is marked by assumptions of responsibilities towards one's family.^19^ In Canada, like in many Western societies, adulthood often tends to coincide with the age of majority and is marked by the assumption of responsibility for oneself in social and economic terms.^20^ Notably, however, cultural values also shift over time, for example, individuals are living with their parents for longer in Canada.^21^

Along with context, in psychosis, symptoms strongly influence functional outcomes, including social, recreational, and independent functioning.^22,23^ We have previously reported that although symptom-related outcomes improved over two years at both sites, Chennai patients had better negative symptom outcomes than Montreal patients.^4^ Accordingly, we hypothesized that social, recreational, and independent functioning after two years’ treatment would also be better in Chennai than Montreal.

This study's primary aim was to investigate differences in social, recreational, and independent functioning over a two-year course between persons with first-episode psychosis in Chennai and Montreal. To our knowledge, no study has examined leisure and recreational functioning and only a handful have examined social and independent functioning among youths with psychosis in LMICs.^24^ Secondly, we determined whether context (Chennai vs. Montreal) predicted these outcomes after accounting for factors known to influence social, independent, and recreational functioning, especially negative symptoms.^25–27^ We also explored certain items (e.g., religious/spiritual activity) on which we expected site differences based on literature and cultural norms.

## Method

### Design and Setting

This paper is a part of a prospective study of patients receiving early intervention for psychosis in the metropolises of Chennai in Tamil Nadu whose literacy rate (82.9%) is higher than the national average, and Montreal in Quebec, Canada's only province whose official language is French.

The study and settings are described in prior publications.^4,5^ Briefly, the Chennai first-episode program, established through a research collaboration with the Montreal site, is in a non-governmental organization, the Schizophrenia Research Foundation (SCARF). The Montreal site comprises two publicly funded early intervention programs in the McGill University network. Both sites have open referral systems, provide free services, and follow a similar treatment protocol based on international guidelines^28,29^ with common core components offered to *all* patients, including case management; lowest effective doses of second-generation antipsychotics; family psychoeducation; close monitoring for two years; and, when needed, other interventions like cognitive-behavioural therapy. Along with these core components, adaptations at the Indian site included cognitive retraining focused on household chores/activities, yoga, etc.

Study procedures were approved by SCARF's Institutional Review Board and McGill University's Research Ethics Board. Adults provided written informed consent, and parental consent was obtained for assenting minors.

### Participants

Participants were persons aged 16–35 receiving services from 2012–2018. Inclusion criteria were a DSM-IV-TR primary diagnosis of schizophrenia-spectrum or affective psychosis; not receiving antipsychotic medication for more than 30 days; and the ability to communicate in Tamil/English in Chennai and French/English in Montreal. Exclusion criteria were having psychosis secondary to a medical condition (e.g., epilepsy) and IQ< 70.

### Measures

All measures were administered by similarly trained staff at both sites, and inter-rater reliability within and between sites was regularly assessed and established to be adequate (details previously reported).^4,30^

#### Social, recreational, and independent functioning

We used a patient-reported outcome measure, the Social Functioning Scale-Early Intervention (SFS-EI),^31^ to assess prosocial (e.g., *going to the movies*), recreation (e.g., *knitting*), and independence-performance (e.g., *cooking*) functioning. We had earlier adapted items to suit diverse contexts (e.g., *church activity* changed to *religious/spiritual activity*) and modernized these subscales (e.g., added *video games*) of the well-established Social Functioning Scale^32^ to create the SFS-EI. The SFS-EI has an *expected independence-performance* subscale (not part of the original scale) that records whether, based on age, family background, and cultural context, each patient was “expected” to perform each item of this subscale. We have published on the SFS-EI's development and psychometric properties, and its translation into French and Tamil.^31^

At baseline (entry into treatment) and at 6, 18, and 24 months after entry, patients completed the SFS-EI, indicating on a 4-point scale (*never*, *rarely*, *sometimes*, and *often*) how frequently they had engaged in each activity in the previous three months. At the same four time-points, clinicians/staff completed the *expected independence-performanc*e subscale.

#### Clinical variables

Diagnoses were established at baseline using the Structured Clinical Interview for DSM-IV-TR Axis I disorders.^33^ Duration of untreated psychosis (DUP), defined as weeks between the onset of the current psychotic episode and the start of antipsychotic treatment, was established using the Circumstances of Onset and Relapse Schedule.^34^ At baseline and Month 3, symptoms were assessed with the Scale for the Assessment of Positive Symptoms,^35^ the Scale for the Assessment of Negative Symptoms,^36^ and the Calgary Depression Scale for Schizophrenia.^37^ At Month 3, using widely used consensus criteria,^38^ early positive remission was defined as scoring ≤2 on each global subscale of hallucinations, delusions, bizarre behaviour, and formal thought disorder; and early negative remission as scoring ≤2 on the flat affect, alogia, avolition, apathy, and anhedonia subscales.

#### Demographic variables

Gender, age at entry, education, and living situation (with family/spouse/in group home vs. living alone/with roommate/homeless) were recorded. Patients’ functional roles (none vs. homemaking/caregiving vs. work/school) were established from the Functional Outcomes Interview,^39^ conducted every six months.

### Statistical Analysis

#### Descriptive analyses

Student's *t* and chi-square tests were used to compare the sites and SFS-EI respondents and non-respondents on baseline characteristics. For each site, we calculated means and standard deviations for the total scores on SFS-EI prosocial, recreation, independence-performance, and expected independence-performance subscales at baseline and months 6, 18, and 24. We also converted the three subscale totals to a percentage of the maximum possible scores.

#### Linear mixed model analyses

We conducted linear mixed model analyses with each of the three subscale total scores as the dependent variable. The main independent variables were time, site, and time-by-site interaction. Demographic and clinical variables known to influence social, recreation, and independent functioning in first-episode psychosis and sociodemographic characteristics on which the two samples differed were included in the models. These were age at entry; gender; living situation; years of education; functional role; primary diagnosis; substance use diagnosis; DUP; positive, negative, and depressive symptom severity; early positive remission status; and early negative remission status.

#### Frequency distribution

The frequencies at which each item of the three subscales was endorsed (*often*, *sometimes*, *rarely*, and *never*) were calculated at Month 6, chosen as the point when most patients were not acutely ill and had received a reasonable duration of treatment.

Using chi-square analyses (*often/sometimes* vs. *rarely/never*), we compared sites on items chosen a priori based on previous literature and cultural norms. We expected:

1. Four prosocial activities (religious/spiritual activity outside the home, formal occasions, visiting relatives in their homes, and being visited by relatives) to be endorsed more in Chennai.^14,15,18^
2. Potentially addictive prosocial and recreational behaviors (taking drugs/alcohol, gambling, and online/video games) to be endorsed more in Montreal, where patients had higher substance use rates.
3. Household independence-performance items (cooking, laundry, and cleaning) to be endorsed more among Chennai women than Chennai men.^40^

To calculate a total for participants not responding to individual item(s) on a subscale, we replaced the missing item with the conservative estimate of zero (i.e., activity *never done*). This was done to a minimum extent with the mean number of imputed items per subscale being less than 1. SPSS v24 was used for analyses. A two-tailed *P-*value of ≤0.05 was considered significant.

## Results

### Descriptive Analyses

The main study included 165 Montreal patients and 168 Chennai patients. Of these, 25 in Montreal and four in Chennai did not fill out the SFS-EI, χ^2^(1) = 17.08, *P* < 0.001, leaving a sample of 140 Montreal and 164 Chennai patients. Montreal respondents had higher baseline negative symptoms compared to Montreal non-respondents, Mean (SD) = 21.15 (11.67) and 15.43 (11.06), respectively, *t*(158) = 2.19, *P* = 0.030. Montreal respondents and non-respondents did not differ in age at entry, gender, marital status, living situation, education, DUP, diagnosis, baseline positive symptoms, or early positive/negative remission.

Compared to Chennai patients, more Montreal patients were single, men, not living with family, younger and had an affective psychotic disorder, comorbid substance use disorder, and higher levels of positive and depressive symptoms (Table 1). This is consistent with previously reported site differences.^4,39^

**Table 1.** Baseline Demographic and Clinical Characteristics.

	Montreal *N* = 140	Chennai *N* = 164	*t* (*df*)	*P* ^a^
Continuous Variables	Mean (SD)	Mean (SD)		
Age at EI entry	24.25 (5.21)	26.50 (5.26)	3.72 (302)	**<0.001**
Years of education	12.37 (2.65)	11.84 (3.91)	1.39 (287)	0.166
Log DUP	1.07 (0.78) Median = 9.0 weeks Range = 0.0–684.3	1.09 (0.60) Median = 11.8 weeks Range = 0.3–223.0	0.22 (219)	0.828
SAPS total score	35.19 (14.74)	20.17 (9.78)	9.92 (211)	**<0.001**
SANS total score	21.15 (11.67)	19.14 (14.53)	1.30 (283)	0.194
CDSS total score	4.36 (3.86)	2.42 (4.35)	3.91 (284)	**<0.001**
*SFS-EI total scores*:				
Prosocial activities	22.90 (11.53)	13.38 (12.16)	5.98 (248)	**<0.001**
Recreation activities	22.18 (9.10)	10.67 (9.34)	9.30 (247)	**<0.001**
Independence-performance	27.76 (9.03)	19.88 (11.06)	6.04 (202)	**<0.001**
Number of expected independence-performance activities^b^	13.44 (3.20)	6.58 (4.35)	14.35 (242)	**<0.001**
Categorical Variables	*N* (valid %)	*N* (valid %)	χ^2^(*df*)	*P*
Men	90 (64.3%)	82 (50.0%)	6.27 (1)	**0.012**
Single	129 (92.8%)	105 (64.0%)	35.44 (1)	**<0.001**
Living with family^c^	106 (78.5%)	138 (97.2%)	22.97 (1)	**<0.001**
Employed/at school	52 (38.8%)	50 (30.9%)	2.05 (1)	0.152
Schizophrenia-spectrum primary diagnosis	95 (68.8%)	146 (90.1%)	21.37 (1)	**<0.001**
Substance use secondary diagnosis	44 (36.1%)	17 (10.5%)	26.98 (1)	**<0.001**

*Note*. CDSS = Calgary Depression Scale for Schizophrenia (possible scores range from 0 to 27); DUP = duration of untreated psychosis; EI = early intervention; SANS = Scale for the Assessment of Negative Symptoms (possible scores range from 0 to 85 after removal of “recreational interests and activities,” attention and global items); SAPS = Scale for the Assessment of Positive Symptoms (possible scores range from 0 to 150); SFS-EI = Social Functioning Scale-Early Intervention (possible score range from 0 to 81 for prosocial activities, 0 to 66 for recreation activities, and 0 to 48 for independence-performance subscales).

^a^ Bold indicates significant differences between Montreal and Chennai.

^b^ Possible number of expected independence-performance activities ranges from 0 to 16.

^c^ Living with family includes living with family/spouse or in a group home. Not living with family includes living alone, with friend/roommate or being homeless. (For the entire sample, only two patients lived in group home and only one was homeless.)

At both sites (Supplementary Figure 1), scores were consistently significantly lower (<40%) for the prosocial and recreation domains than for the independence-performance domain (40–65%), linear mixed model *F*(2, 891) = 366, *P* < 0.001. Prosocial and recreation scores, however, did not differ from each other.

### Linear Mixed Model Analyses

#### Prosocial activities

**Figure 1.**
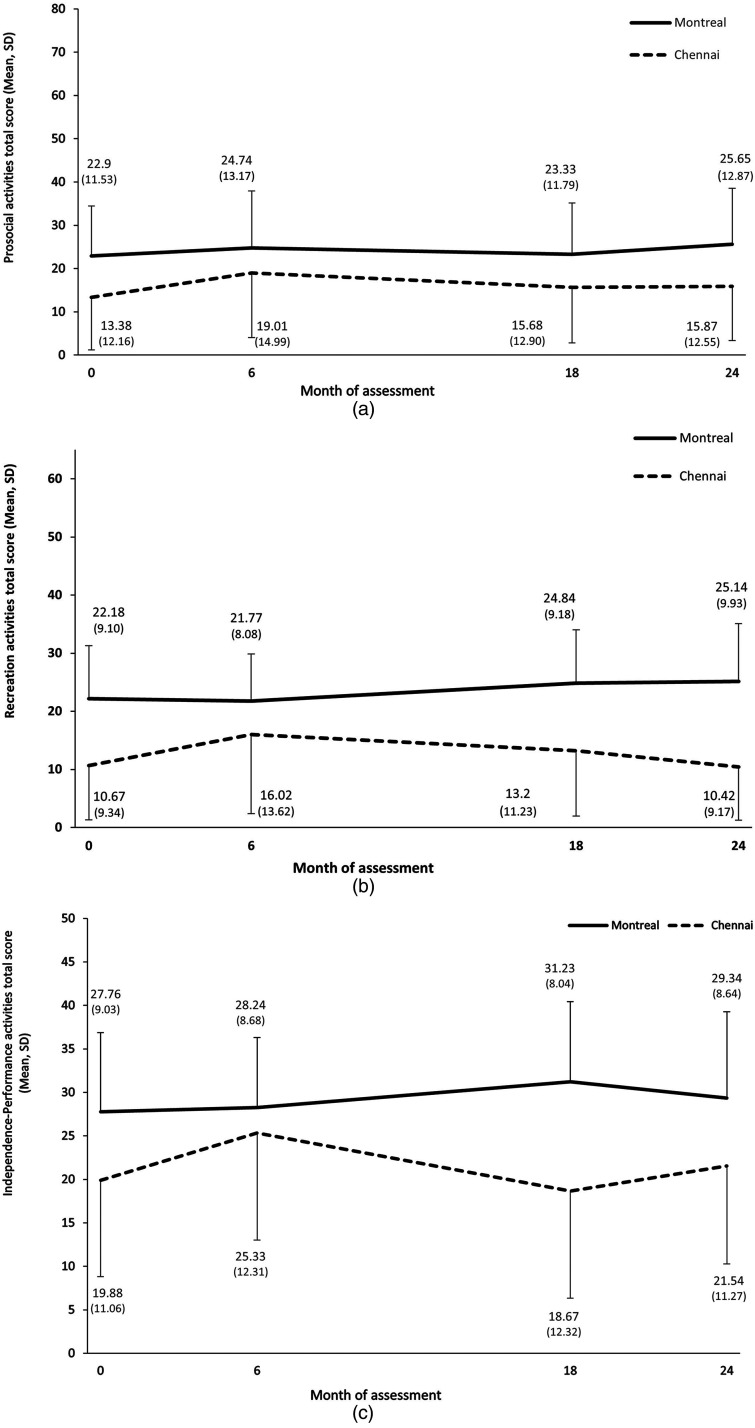
(a) Prosocial activities in Montreal and Chennai over the two-year follow-up. Prosocial activities possible total score ranges from 0 to 81. (b) Recreation activities in Montreal and Chennai over the two-year follow-up. Recreation activities possible total score ranges from 0 to 66. (c) Independence-performance activities in Montreal and Chennai over the two-year follow-up. Independence-performance activities possible total score ranges from 0 to 48.

The maximum possible total for prosocial activities is 81. At baseline, Montreal patients had a significantly higher score than Chennai patients, Mean (SD) = 22.90 (11.53) and 13.38 (12.16), respectively, *t*(248) = 5.98, *P* < 0.001. Linear mixed model analysis (Table 2) revealed that this site difference was sustained over the two-year follow-up, *F*(1, 346) = 5.13, *P* = 0.024. Prosocial activities scores did not change from baseline to Month 24 at either site, *F*(1, 265) = 0.44, *P* = 0.510 (Figure 1a). Individuals who were younger, *F*(1, 192) = 7.63, *P* = 0.006, more educated, *F*(1, 167) = 8.02, *P* = 0.005, or had lower negative symptoms, *F*(1, 170) = 5.78, *P* = 0.017, had higher prosocial functioning scores.

**Table 2.** Linear Mixed Model with Prosocial Activities Total Score as the Outcome.

	Prosocial Activities Total Score						
	*B*	*SE*	*df*	*t*	*P* ^a^	95% CI for *B*	
						Lower Bound	Upper Bound
Time (month)	0.01	0.06	155	0.09	0.931	−0.11	0.12
Montreal (Chennai is reference)	5.15	2.27	346	2.27	**0.024** ^c^	0.68	9.62
Time (month) × Montreal (Chennai is reference)	0.07	0.12	264	0.57	0.566	−0.16	0.29
Age at EI entry	−0.35	0.13	192	−2.76	**0.006** ^c^	−0.61	−0.10
Men (women is reference)	−0.25	1.50	218	−0.17	0.869	−3.20	2.71
Living with family at baseline	−4.41	2.34	224	−1.88	0.061	−9.02	0.20
Education (years)	0.52	0.18	167	2.83	**0.005** ^c^	0.16	0.88
Role^b^: homemaker/caregiver (none is reference)	−1.35	1.98	279	−0.68	0.497	−5.24	2.55
Role: employed/student (none is reference)	−1.13	1.24	588	−0.91	0.364	−3.56	1.31
Schizophrenia spectrum primary diagnosis (affective psychosis is reference)	−1.48	1.75	197	−0.85	0.398	−4.93	1.97
Substance use secondary diagnosis	2.37	1.75	219	1.35	0.177	−1.08	5.81
Log DUP	−1.21	0.98	183	−1.24	0.215	−3.14	0.71
SAPS total score at baseline	0.04	0.05	196	0.74	0.460	−0.06	0.14
SANS total score at baseline	−0.11	0.05	170	−2.40	**0.017** ^c^	−0.21	−0.02
Early positive remission (Months 1 to 3)	0.46	1.70	194	0.27	0.785	−2.89	3.82
Early negative remission (Months 1 to 3)	0.40	1.79	201	0.22	0.823	−3.13	3.93
CDSS total score at baseline	−0.28	0.17	179	−1.70	0.090	−0.61	0.04

*Note:* CDSS = Calgary Depression Scale for Schizophrenia; DUP = duration of untreated psychosis; EI = early intervention; SANS = Scale for the Assessment of Negative Symptoms; SAPS = Scale for the Assessment of Positive Symptoms.

^a^ Bold is significant.

^b^ Role = employed/student, homemaker/caregiver, or none over the two-year follow-up.

^c^ The Montreal site, younger age, higher education, and lower negative symptoms are significantly associated with higher prosocial activities; Type III test *F*(1, 346) = 5.13, *P* = 0.024, Type III test *F*(1, 192) = 7.63, *P* = 0.006, Type III test *F*(1, 167) = 8.02, *P* = 0.005, and Type III test *F*(1, 170) = 5.78, *P* = 0.017, respectively.

#### Recreation activities

The maximum possible total for recreation activities is 66. At baseline, Montreal scored higher than Chennai, Mean (SD) = 22.18 (9.10) and 10.67 (9.34), respectively, *t*(247) = 9.30, *P* < 0.001. This site difference was maintained over the two-year follow-up, *F*(1, 356) = 13.04, *P* < 0.001. The main effect of time was not significant, *F*(1, 289) = 1.19, *P* = 0.276. However, site-by-time interaction showed that Montreal patients scored more in recreation activities over time than Chennai patients, *F*(1, 287) = 7.44, *P* = 0.007 (Table 3; Figure 1b). Again, individuals who were more educated, *F*(1, 166) = 7.50, *P* = 0.007, and had lower negative symptoms reported higher levels of recreation activities, *F*(1, 168) = 4.68, *P* = 0.032.

**Table 3.** Linear Mixed Model with Recreation Activities Total Score as the Outcome.

	Recreation Activities Total Score						
	*B*	*SE*	*df*	*t*	*P^a^*	95% CI for *B*	
						Lower Bound	Upper Bound
Time (month)	−0.08	0.05	170	−1.65	0.102	−0.17	0.02
Montreal (Chennai is reference)	6.76	1.87	356	3.61	**0.000** ^c^	3.08	10.45
Time (month) × Montreal (Chennai is reference)	0.26	0.10	287	2.73	**0.007** ^d^	0.07	0.45
Age at EI entry	−0.17	0.10	188	−1.65	0.100	−0.38	0.03
Men (women is reference)	0.48	1.24	220	0.39	0.699	−1.96	2.92
Living with family at baseline	−1.22	1.91	220	−0.64	0.523	−4.99	2.55
Education (years)	0.41	0.15	166	2.74	**0.007** ^c^	0.12	0.71
Role^b^: homemaker/caregiver (none is reference)	−1.63	1.64	271	−0.99	0.321	−4.85	1.60
Role: employed/student (none is reference)	−1.34	1.06	556	−1.26	0.209	−3.43	0.75
Schizophrenia spectrum primary diagnosis (affective psychosis is reference)	−1.38	1.44	199	−0.96	0.338	−4.22	1.46
Substance use secondary diagnosis	1.41	1.45	223	0.97	0.332	−1.44	4.26
Log DUP	0.06	0.80	179	0.08	0.936	−1.51	1.64
SAPS total score at baseline	0.01	0.04	197	0.29	0.772	−0.07	0.09
SANS total score at baseline	−0.08	0.04	168	−2.16	**0.032** ^c^	−0.16	−0.01
Early positive remission (Months 1 to 3)	1.15	1.39	191	0.83	0.409	−1.59	3.89
Early negative remission (Months 1 to 3)	0.13	1.47	202	0.09	0.929	−2.78	3.04
CDSS total score at baseline	−0.07	0.14	177	−0.54	0.589	−0.34	0.19

*Note:* CDSS = Calgary Depression Scale for Schizophrenia; DUP = duration of untreated psychosis; EI = early intervention; SANS = Scale for the Assessment of Negative Symptoms; SAPS = Scale for the Assessment of Positive Symptoms.

^a^ Bold is significant.

^b^ Role = employed/student, homemaker/caregiver, or none over the two-year follow-up.

^c^ The Montreal site, higher education, and lower negative symptoms are significantly associated with higher recreation activities; Type III test *F*(1, 356) = 13.04, *P* < 0.001, Type III test *F*(1, 166) = 7.50, *P* = 0.007, and Type III test *F*(1, 168) = 4.68, *P* = 0.032, respectively.

^d^ Recreation activities in Montreal increases by time more than in Chennai; Type III test *F*(1, 287) = 7.44, *P* = 0.007.

#### Independence-performance activities

The maximum possible total for independence-performance is 48. At baseline, Montreal had higher scores than Chennai, Mean (SD) = 27.76 (9.03) and 19.88 (11.06), respectively, *t*(202) = 6.04, *P* < 0.001. Linear mixed model analysis (Table 4; Figure 1c) showed that this site difference stayed significant over the two-year follow-up, *F*(1, 338) = 7.29, *P* = 0.007. Independence-performance did not change over time at either site. Education significantly predicted higher independence-performance, *F*(1, 170) = 5.88, *P* = 0.016.

**Table 4.** Linear Mixed Model with Independence-Performance Total Score as the Outcome.

	Independence-Performance						
	*B*	*SE*	*df*	*t*	*P* ^a^	95% CI for *B*	
						Lower Bound	Upper Bound
Time (month)	−0.06	0.05	201	−1.26	0.210	−0.16	0.03
Montreal (Chennai is reference)	5.38	1.99	338	2.70	**0.007** ^c^	1.46	9.31
Time (month) × Montreal (Chennai is reference)	0.19	0.10	314	1.92	0.056	0.00	0.38
Age at EI entry	0.03	0.11	190	0.27	0.784	−0.19	0.26
Men (women is reference)	−0.41	1.33	218	−0.31	0.758	−3.04	2.22
Living with family at baseline	−3.70	2.06	216	−1.79	0.075	−7.76	0.37
Education (years)	0.40	0.16	170	2.42	**0.016** ^c^	0.07	0.72
Role^b^: homemaker/caregiver (none is reference)	1.24	1.76	274	0.71	0.481	−2.23	4.71
Role: employed/student (none is reference)	−0.06	1.13	547	−0.05	0.957	−2.28	2.16
Schizophrenia spectrum primary diagnosis (affective psychosis is reference)	1.69	1.55	197	1.09	0.277	−1.37	4.75
Substance use secondary diagnosis	0.64	1.56	222	0.41	0.682	−2.44	3.72
Log DUP	−0.96	0.86	179	−1.11	0.269	−2.66	0.75
SAPS total score at baseline	0.04	0.04	198	0.97	0.332	−0.04	0.13
SANS total score at baseline	−0.07	0.04	171	−1.79	0.076	−0.16	0.01
Early positive remission (Months 1 to 3)	−0.25	1.50	192	−0.17	0.868	−3.22	2.72
Early negative remission (Months 1 to 3)	1.63	1.59	202	1.02	0.308	−1.51	4.77
CDSS total score at baseline	−0.16	0.15	180	−1.10	0.273	−0.45	0.13

*Note:* CDSS = Calgary Depression Scale for Schizophrenia; DUP = duration of untreated psychosis; EI = early intervention; SANS = Scale for the Assessment of Negative Symptoms; SAPS = Scale for the Assessment of Positive Symptoms.

^a^ Bold is significant.

^b^ Role = employed/student, homemaker/caregiver, or none over the two-year follow-up.

^c^ The Montreal site; Type III test *F*(1, 338) = 7.29, *P* = 0.007 and higher education; Type III test *F*(1, 170) = 5.88, *P* = 0.016 are significantly associated with higher independence-performance total score.

When the variable “expected independence-performance” was added to the model (Table 5), it significantly predicted actual independence-performance, *F*(1, 296) = 15.10, *P* < 0.001, and the site effect was no longer significant, *F*(1, 268) = 1.64, *P* = 0.202. Time remained not significant, *F*(1, 213) = 3.28, *P* = 0.072. Higher education, *F*(1, 157) = 4.99, *P* = 0.027, and lower baseline depression, *F*(1, 183) = 4.88, *P* = 0.028, were associated with higher independence-performance.

**Table 5.** Linear Mixed Model with Independence-Performance Total Score as the Outcome and Number of Expected Independence-Performance Activities as a Predictor.

	Independence-Performance						
	*B*	*SE*	*df*	*t*	*P* ^a^	95% CI for *B*	
						Lower Bound	Upper Bound
Time (month)	0.48	0.26	198	1.82	0.070	−0.04	0.99
Montreal (Chennai is reference)	3.29	2.57	268	1.28	0.202	−1.78	8.36
Time (month) × Montreal (Chennai is reference)	−0.19	0.41	208	−0.45	0.651	−1.00	0.63
Age at EI entry	−0.01	0.14	170	−0.11	0.914	−0.28	0.26
Men (women is reference)	1.15	1.59	178	0.72	0.471	−2.00	4.30
Living with family at baseline	−3.26	2.33	182	−1.40	0.164	−7.87	1.34
Education (years)	0.44	0.20	157	2.23	**0.027** ^c^	0.05	0.83
Role^b^: homemaker/caregiver (none is reference)	2.23	2.15	194	1.03	0.302	−2.02	6.48
Role: employed/student (none is reference)	−1.58	1.46	287	−1.08	0.280	−4.45	1.29
Schizophrenia spectrum primary diagnosis (affective psychosis is reference)	1.33	1.74	172	0.77	0.444	−2.10	4.77
Substance use secondary diagnosis	0.82	1.83	193	0.45	0.655	−2.79	4.43
Log DUP	−0.74	0.98	165	−0.76	0.450	−2.68	1.19
SAPS total score at baseline	0.09	0.05	179	1.63	0.106	−0.02	0.19
SANS total score at baseline	−0.08	0.05	155	−1.61	0.108	−0.17	0.02
Early positive remission (Months 1 to 3)	−0.54	1.76	180	−0.31	0.760	−4.02	2.94
Early negative remission (Months 1 to 3)	2.98	1.85	177	1.61	0.108	−0.66	6.63
CDSS total score at baseline	−0.39	0.18	183	−2.21	**0.028** ^c^	−0.73	−0.04
Number of expected independence-performance activities at baseline and Month 6.	0.63	0.16	296	3.89	**0.000** ^c^	0.31	0.94

*Note:* CDSS = Calgary Depression Scale for Schizophrenia; DUP = duration of untreated psychosis; EI = early intervention; SANS = Scale for the Assessment of Negative Symptoms; SAPS = Scale for the Assessment of Positive Symptoms.

^a^ Bold is significant.

^b^ Role = employed/student, homemaker/caregiver, or none over the two-year follow-up.

^c^ Higher education, Type III test *F*(1, 157) = 4.99, *P* = 0.027; lower baseline depressive symptoms, *F*(1, 183) = 4.88, *P* = 0.028; and larger number of expected independence-performance activities, *F*(1, 296) = 15.10, *P* < 0.001 are significantly associated with higher actual independence-performance total score.

#### Expected independence-performance

**Figure 2.**
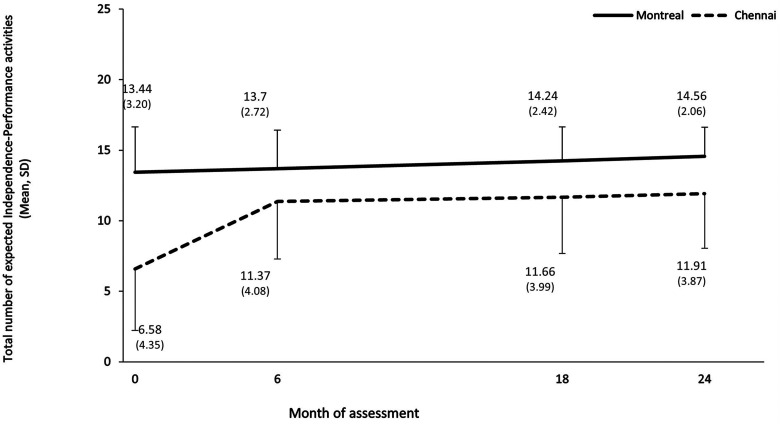
Number of expected independence-performance activities in Montreal and Chennai over the two-year follow-up. Possible number of expected independence-performance activities ranges from 0 to 16.

At baseline, more independence-performance activities were expected from Montreal than Chennai patients, Mean (SD) = 13.44 (3.20) and 6.58 (4.35), respectively, *t*(242) = 14.35, *P* < 0.001 (Table 1). Linear mixed model analyses (Figure 2; Table 6) revealed that this significant site difference persisted over the two-year follow-up, *F*(1, 280) = 60.59, *P* < 0.001. Over time, expectations about independence-performance increased significantly, *F*(1, 184) = 56.99, *P* < 0.001, particularly in Chennai, *F*(1, 183) = 21.77, *P* < 0.001. Finally, expectations about independence-performance were higher from individuals who were more educated, *F*(1, 175) = 7.40, *P* = 0.007; worked/were in school (vs. those not in employment or education), *F*(2, 444) = 4.84, *P* = 0.008; and had lower negative symptoms, *F*(1, 172) = 4.21, *P* = 0.042.

**Table 6.** Linear Mixed Model with Number of Expected Independence-Performance Activities as the Outcome.

	Number of Expected Independence-Performance Activities						
	*B*	*SE*	*df*	*t*	*P* ^a^	95% CI for *B*	
						Lower Bound	Upper Bound
Time (month)	0.18	0.02	167	9.63	**0.000** ^c^	0.14	0.21
Montreal (Chennai is reference)	5.37	0.69	280	7.78	**0.000** ^c^	4.02	6.73
Time (month) × Montreal (Chennai is reference)	−0.14	0.03	183	−4.67	**0.000** ^d^	−0.19	−0.08
Age at EI entry	0.05	0.04	181	1.26	0.209	−0.03	0.13
Men (women is reference)	−0.73	0.48	209	−1.51	0.132	−1.68	0.22
Living with family at baseline	0.25	0.70	182	0.36	0.721	−1.12	1.62
Education (years)	0.17	0.06	175	2.72	**0.007** ^c^	0.05	0.30
Role^b^: homemaker/caregiver (none is reference)	0.43	0.63	313	0.68	0.499	−0.81	1.66
Role: employed/student (none is reference)	1.09	0.35	653	3.11	**0.002** ^c^	0.40	1.77
Schizophrenia spectrum primary diagnosis (affective psychosis is reference)	0.53	0.53	179	1.01	0.314	−0.51	1.58
Substance use secondary diagnosis	0.69	0.53	184	1.30	0.196	−0.36	1.75
Log DUP	−0.29	0.31	179	−0.93	0.354	−0.89	0.32
SAPS total score at baseline	0.00	0.02	179	0.03	0.973	−0.03	0.03
SANS total score at baseline	−0.03	0.02	172	−2.05	**0.042** ^c^	−0.06	0.00
Early positive remission (Months 1 to 3)	−0.20	0.53	179	−0.38	0.702	−1.26	0.85
Early negative remission (Months 1 to 3)	−0.02	0.57	186	−0.03	0.979	−1.14	1.11
CDSS total score at baseline	0.09	0.05	192	1.73	0.085	−0.01	0.20

*Note:* CDSS = Calgary Depression Scale for Schizophrenia; DUP = duration of untreated psychosis; EI = early intervention; SANS = Scale for the Assessment of Negative Symptoms; SAPS = Scale for the Assessment of Positive Symptoms.

^a^ Bold is significant.

^b^ Role = employed/student, homemaker/caregiver, or none over the two-year follow-up.

^c^ Time, Montreal site, higher education, and lower negative symptoms are significantly associated with number of expected independence-performance activities; Type III test *F*(1, 184) = 56.99, *P* < 0.001, Type III test *F*(1, 280) = 60.59, *P* < 0.001, Type III test *F*(1, 175) = 7.40, *P* = 0.007, and Type III test *F*(1, 172) = 4.21, *P* = 0.042, respectively. Not working, studying, homemaking, or caregiving is associated with a smaller number of expected independence-performance activities as compared to working/studying; Type III test *F*(2, 444) = 4.84, *P* = 0.008.

^d^ The number of expected independence-performance activities increases by time in Chennai more than in Montreal; Type III test *F*(1, 183) = 21.77, *P* < 0.001.

### Frequency Distribution of Individual Items

The frequencies at which the items of the three subscales were endorsed in Chennai and Montreal are presented in Supplementary Figures 2(a) to (c) and Supplementary Tables 1(a) to (f).

As predicted, more Chennai patients did *religious/spiritual activities outside the home*, 54.5% vs.26.3%, respectively, χ^2^(1) = 17.93, *P* < 0.001 and attended *formal occasions* (e.g., weddings), 29.3% vs. 13.1%, respectively, χ^2^(1) = 8.30, *P* = 0.004, more frequently than Montreal patients. There were no significant site differences in the extent to which patients endorsed *visiting relatives*, χ^2^(1) = 1.95, *P* = 0.163, or *being visited by relatives*, χ^2^(1) = 3.41, *P* = 0.065.

As predicted, more Montreal patients were likely to be *taking drugs/alcohol with others*, 29.3% vs. 7.3% respectively, χ^2^(1) = 18.67, *P* < 0.001, but not *gambling at a casino/video terminal*, χ^2^(1) = 1.70, *P* = 0.192, compared to Chennai patients. Contrary to expectations, Montreal patients did not endorse *using drugs/alcohol alone*, *online gambling*, and/or *playing video or online games* more frequently than Chennai patients.

For household independence-performance items, as expected, Chennai women were likelier to engage *often/sometimes* in *cooking*, 68.3% women vs. 25.4% men, χ^2^(1) = 22.78, *P* < 0.001; *laundry*, 81.7% women vs. 49.2% men, χ^2^(1) = 14.24, *P* < 0.001; and *cleaning*, 72.9% women vs. 34.9% men, χ^2^(1) = 17.64, *P* < 0.001, compared to Chennai men. This pattern prevailed in Montreal for *cooking*, 73.5% women vs. 52.3% men, χ^2^(1) = 4.18, *P* = 0.041, and *laundry*, 82.4% women vs. 60.0% men, χ^2^(1) = 5.10, *P* = 0.024, but not *cleaning*. Site differences on these items were consistent with those on the overall subscale, with Montreal participants more frequently engaging in cooking, χ^2^(1) = 3.86, *P* = 0.049, and cleaning, χ^2^(1) = 4.71, *P* = 0.030 (but not laundry).

## Discussion

To our knowledge, this is the first cross-cultural investigation of social, recreational, and independent functioning in early intervention for psychosis, and the first examination of leisure and recreational functioning in first-episode psychosis in an LMIC.

At both sites, prosocial, recreation, and independence-performance functioning was not high and did not improve. Earlier studies^27,41^ (with one exception^42^) too reported no change in these SFS domains six months to a year after early intervention. We found stability in these outcomes over a longer (two-year) period in two distinct contexts. In these same Chennai and Montreal samples, both positive and negative symptoms significantly improved.^4^ These findings thus underscore the *symptom-functioning gap* in psychotic disorders^43,44^ and highlight the need for interventions targeting functional improvements.^45^

Contrary to the hypothesis and despite their better negative symptom outcomes, Chennai patients had lower scores on all three domains than Montreal patients. As theorized,^46,47^ negative symptoms significantly predicted all three domains even after accounting for site. Nonetheless, site differences persisted, suggesting that various contextually and culturally shaped influences may be at play.

### Contextually and Culturally Shaped Influences

Notions of leisure and the importance given to it are influenced by cultural context.^48^ In keeping with the degree of importance accorded to autonomy,^12^ leisure and recreation may be deemed more central to quality of life and well-being in HICs like Canada than in LMICs like India.^49,50^ Furthermore, time and financial constraints may impede leisure and recreation in India.^51,52^

In keeping with the importance accorded to embeddedness,^12^ Chennai patients frequently interacted with family or other social networks and visited places of worship. They also did more activities *with* their families. In post hoc analyses, twice as many Chennai patients (45.5%) as Montreal ones (21.3%) strongly agreed with the statement, “*My family and I did activities together*,” χ^2^(5) = 18.5, *P* < 0.01. More Chennai youths lived with their families. A higher value is also placed on familial interactions and obligations in India.^4,5,14,15,53^ However, as previously reported for Indian adolescents, some patients may have felt pressured to socialize with their families.^54^ What bears examination is whether Indian youth with psychosis may be additionally pressured to restrict socialization to their families because of stigma, discrimination, and anticipated shunning by outsiders.^55^ Across contexts, stigma and anticipated and experienced discrimination impede the full participation of persons with psychosis.^56^

The site difference with respect to visiting religious places bespeaks both the importance of religion in India^16,18,57^ and the receding role of the church and religion in Quebec, a highly secularized society.^58^

Strikingly, the lower independence-performance of Chennai patients was nearly entirely attributable to the varying level of expectations of independence-performance. This variable has not been considered thus far in independent functioning research in psychosis. Compared to Montreal, a significantly lower level of expectation was placed on Chennai patients. This could be explained by varying norms about the age at which individuals are expected to “launch out” or assume responsibility for independent living.^59^ It could also relate to cultural norms around lowering expectations from the unwell and around how much responsibility caregivers assume or are entrusted with.^60^ That they increased over time suggests that expectations around independence-performance are also influenced by clinicians’ (and families’) perceptions about illness severity, which decreases over time. Economic realities may also hinder independent functioning, especially in India. Consistent with often-reported trends, findings suggest that across contexts, women bear more responsibility for household-related independent functioning.^40,61,62^

### Role of Education

The positive relationship between education and amount of leisure activity has been well documented in general populations in HICs and LMICs.^63,64^ This study extends this finding to an early psychosis population and shows that education remains beneficial for prosocial, recreation, and independent functioning after accounting for site, illness factors, and expectations.

There were fewer expectations about independent functioning from patients who were neither in education, employment, or training nor homemakers, even after accounting for factors like symptoms. This association bears investigation with larger samples as lowered expectations may be part of the vicious cycle of functional disability and social exclusion among those who are not in education, employment, or training. Overall, however, being employed/in school did not independently predict the three subscales, a finding that is inconsistent with previous schizophrenia research.^26,32,65^

### Implications

Evidence for the benefits of prosocial and leisure activities in the general population is mounting in the West and in LMICs.^50,52^ This study's findings of modest performance in these domains and in independent functioning are therefore disconcerting. Our findings strengthen the argument^45^ that facilitating sustained negative symptom remission will improve functional outcomes in psychosis *across* contexts. They also highlight the need for interventions targeting functional domains given the symptom-functioning gap.

The detailed information (including activity type) on prosocial, recreation, and independent functioning over a two-year period of patients with psychosis that this study provides is valuable for addressing these areas. For instance, sedentary activities like watching TV were frequently endorsed at both sites. Such activities, being home-based or private,^66^ may be seen as safer. Conversely, active pursuits like walking/jogging/gym were infrequently endorsed and may need encouragement, given the high rates of cardiometabolic problems in psychosis.^67^

Uniquely, this study highlights the importance of expectations about role functioning, a potentially malleable factor that could be targeted to improve functioning.

### Strengths

Adding a subscale to assess expected independence-performance yielded sophisticated insights into how expectations mediate cross-cultural differences in functioning. Furthermore, while the SFS has been used in early psychosis in HICs,^25,27,41^ this study with its repeated assessments over two years, goes well beyond the six-month follow-up period in most studies. A single study^26^ found significant improvement four to six years after the first hospitalization, suggesting that improving these outcomes may require sustained treatment. More research with longer follow-ups is warranted.

### Limitations

Having been recruited from publicly funded programs serving specified catchments, Montreal patients may be more representative of a treated incidence sample. The Chennai site is a primarily outpatient, non-geographically restricted facility. Its patients came from a wider-spread population base, but few entered treatment through emergency services. This may explain inter-site variation in sociodemographic characteristics. However, site differences in the three studied domains persisted after accounting for these characteristics.

The method used for imputing missing items may have over-estimated the proportion of items endorsed as *never* being done, particularly in Montreal. This might have been especially problematic for risk-taking behaviors such as alcohol/drug use. Nonetheless, imputation was done sparingly.

Measures like the SFS may have limited ecological validity.^68^ Future research should therefore exploit technology to capture real-time, experience-based functioning.

This study involved two specific contexts, and its findings cannot be generalized to *all* HICs-LMICs or even across Canada and India. The Montreal sample may have included subgroups (e.g., visible minority) with varying expectations/values and stigma perceptions associated with functioning, which were not analysed. Data collection was completed in 2018. Subsequent social and technological changes and the pandemic may mean that the findings may not fully reflect present-day trends. For example, patients at both sites may now spend more time online.

## Conclusion

This study provides evidence for context shaping prosocial, recreation, and independent functioning in psychosis and for cross-cultural variations in functioning being partially explained by patients being expected to do more or less. Future research should examine the impacts of social, recreational, and independent functioning on recovery, loneliness,^69^ and boredom,^11,70^ and the *meaning* of these domains for youth using qualitative and other ethnographically informed approaches. Irrespective of context, there is a need for interventions targeting these domains.

## Supplemental Material

sj-docx-1-cpa-10.1177_07067437231153796 - Supplemental material for Context and Expectations Matter: Social, Recreational, and Independent Functioning among Youth with Psychosis in Chennai, India and Montreal, Canada Click here for additional data file. Supplemental material, sj-docx-1-cpa-10.1177_07067437231153796 for Context and Expectations Matter: Social, Recreational, and Independent Functioning among Youth with Psychosis in Chennai, India and Montreal, Canada by Srividya N. Iyer, Thara Rangaswamy, Sally Mustafa, Nicole Pawliuk, Greeshma Mohan, Ridha Joober, Norbert Schmitz, Howard Margolese, Ramachandran Padmavati and Ashok Malla in The Canadian Journal of Psychiatry
