# Supplementary material for: Context and Expectations Matter: Social, Recreational, and Independent Functioning among Youth with Psychosis in Chennai, India and Montreal, Canada
Source: Can J Psychiatry. 2023 Feb 6;68(10):766–79. doi: 10.1177/07067437231153796 (PMC10517650; doi:10.1177/07067437231153796)
Supplement: sj-docx-1-cpa-10.1177_07067437231153796 - Supplemental material for Context and Expectations Matter: Social, Recreational, and Independent Functioning among Youth with Psychosis in Chennai, India and Montreal, Canada [file sj-docx-1-cpa-10.1177_07067437231153796.docx]

**Supplementary information**

**Context and expectations matter: Social, recreational and independent functioning among youth with psychosis in Chennai, India and Montreal, Canada**, *Canadian Journal of Psychiatry,* Iyer*, Rangaswamy, Mustafa, Pawliuk, Mohan, Joober, Schmitz, Margolese, Padmavati and Malla.

*Corresponding author: [srividya.iyer@mcgill.ca](mailto:srividya.iyer@mcgill.ca); Department of Psychiatry, McGill University, Montreal, Canada; Prevention and Early Intervention Program for Psychosis (PEPP-Montreal), Douglas Mental Health University Institute, Montreal, Canada.

| **Item** | **Title** | **Page** |
| --- | --- | --- |
| Supplementary Figure 1 | **Social Functioning Scale - Early Intervention percent scores** broken by subscale (prosocial, independence-performance and recreation) and site (Montreal and Chennai) **over the two-year follow-up** | 2 |
| Supplementary Figure 2a | Frequency of **prosocial activities by site** at Month 6 of follow-up | 3 |
| Supplementary Figure 2b | Frequency of **recreation activities by site** at Month 6 of follow-up | 4 |
| Supplementary Figure 2c | Frequency of **independence-performance activities by site** at Month 6 of follow-up | 5 |
| Supplementary Table 1a | Frequency of **prosocial activities in Montreal** at Month 6 of follow-up | 6 |
| Supplementary Table 1b | Frequency of **prosocial activities in Chennai** at Month 6 of follow-up | 7 |
| Supplementary Table 1c | Frequency of **recreation activities in Montreal** at Month 6 of follow-up | 8 |
| Supplementary Table 1d | Frequency of **recreation activities in Chennai** at Month 6 of follow-up | 9 |
| Supplementary Table 1e | Frequency of **independence-performance activities in Montreal** at Month 6 of follow-up | 10 |
| Supplementary Table 1f | Frequency of **independence-performance activities in Chennai** at Month 6 of follow-up | 11 |

**Supplementary Figure 1. Social Functioning Scale - Early Intervention percent scores broken by subscale (prosocial, independence-performance and recreation) and site (Montreal and Chennai) over the two-year follow-up**

Montreal
Chennai

Montreal
Chennai

Montreal
Chennai

Montreal
Chennai

Montreal
Chennai

Montreal
Chennai

Montreal
Chennai

Montreal
Chennai

Montreal
Chennai

Montreal
Chennai

Montreal
Chennai

Montreal
Chennai

Montreal
Chennai

Montreal
Chennai

Montreal
Chennai

Montreal
Chennai

Montreal
Chennai

Montreal
Chennai

Montreal
Chennai

Montreal
Chennai

Montreal
Chennai

Montreal
Chennai

Montreal
Chennai

Montreal
Chennai

Montreal
Chennai

Montreal
Chennai

Montreal
Chennai

Note: Twenty-five patients in Montreal and 10 in Chennai chose to list “other” prosocial activities. Most open-ended responses were, however, already part of the scale, albeit in other subscales. Novel examples given in Montreal were “chilling out,” “volunteering,” and “coaching a sport.” Six patients in Chennai mentioned “travelling in public transport,” and others were work- or shopping-related.

**Supplementary Figure 2a. Frequency of prosocial activities by site at Month 6 of follow-up**

Montreal
Chennai

Montreal
Chennai

Montreal
Chennai

Montreal
Chennai

Montreal
Chennai

Montreal
Chennai

Montreal
Chennai

Montreal
Chennai

Montreal
Chennai

Montreal
Chennai

Montreal
Chennai

Montreal
Chennai

Montreal
Chennai

Montreal
Chennai

Montreal
Chennai

Montreal
Chennai

Montreal
Chennai

Montreal
Chennai

Montreal
Chennai

Montreal
Chennai

Montreal
Chennai

Montreal
Chennai

Note: Fourteen Montreal patients and none in Chennai chose to list “other” recreation activities, many of which were already part of the scale. Novel responses included “writing poetry,” “dance” and “online training for work.”

**Supplementary Figure 2b. Frequency of recreation activities by site at Month 6 of follow-up**

Montreal
Chennai

Montreal
Chennai

Montreal
Chennai

Montreal
Chennai

Montreal
Chennai

Montreal
Chennai

Montreal
Chennai

Montreal
Chennai

Montreal
Chennai

Montreal
Chennai

Montreal
Chennai

Montreal
Chennai

Montreal
Chennai

Montreal
Chennai

Montreal
Chennai

Montreal
Chennai

**Supplementary Figure 2c. Frequency of independence-performance activities at Month 6 of follow-up**

**Supplementary Table 1a. Frequency of prosocial activities in Montreal at Month 6 of follow-up**

| **No.** | **Prosocial activity** | **Frequency (%)** | | | |
| --- | --- | --- | --- | --- | --- |
|  |  | **Often** | **Sometimes** | **Rarely** | **Never** |
| 1 | Going to the cinema/movies | 12.1 | 38.4 | 19.2 | 30.3 |
| 2 | Theatre/concert | 0 | 21.2 | 19.2 | 59.6 |
| 3 | Watching an indoor sport in person, not on TV (e.g., hockey, basketball) | 5.1 | 10.1 | 11.1 | 73.7 |
| 4 | Watching an outdoor sport in person, not on TV (e.g., soccer, baseball) | 1.0 | 11.1 | 18.2 | 69.7 |
| 5 | Art gallery/museum | 2.0 | 16.2 | 22.2 | 59.6 |
| 6 | Exhibition | 2.0 | 15.2 | 21.2 | 61.6 |
| 7 | Visiting places of interest (e.g., beach, parks) | 19.2 | 35.4 | 18.2 | 27.3 |
| 8 | Meetings, talks, etc. (outside the house) | 24.2 | 40.4 | 21.2 | 14.1 |
| 9 | Taking a class (outside the house) | 20.2 | 15.2 | 10.1 | 54.5 |
| 10 | Visiting relatives in their homes | 21.2 | 35.4 | 19.2 | 24.2 |
| 11 | Being visited by relatives | 19.2 | 25.3 | 24.2 | 31.3 |
| 12 | Visiting/hanging out with friends (including boyfriends/girlfriends) | 41.4 | 23.2 | 20.2 | 15.2 |
| 13 | Parties | 9.1 | 23.2 | 31.3 | 36.4 |
| 14 | Formal occasions (e.g., wedding, baptism) | 3.0 | 10.1 | 24.2 | 62.6 |
| 15 | Disco/nightclub | 3.0 | 13.1 | 23.2 | 60.6 |
| 16 | Club/society | 6.1 | 9.1 | 13.1 | 71.7 |
| 17 | Playing an indoor sport | 7.1 | 16.2 | 19.2 | 57.6 |
| 18 | Playing an outdoor sport | 6.1 | 18.2 | 17.2 | 58.6 |
| 19 | Pub/bar | 5.1 | 23.2 | 24.2 | 47.5 |
| 20 | Eating at a restaurant/outside the house | 22.2 | 46.5 | 19.2 | 12.1 |
| 21 | Religious/spiritual activity outside the home | 7.1 | 19.2 | 14.1 | 59.6 |
| 22 | Video chat (e.g., Skype)/online chatting (e.g., MSN) with relatives | 13.1 | 17.2 | 13.1 | 56.6 |
| 23 | Video chat (e.g., Skype)/online chatting (e.g., MSN) with friends | 13.1 | 20.2 | 17.2 | 49.5 |
| 24 | Working out at the gym | 9.1 | 17.2 | 21.2 | 52.5 |
| 25 | Group activity at the gym (e.g., zumba, spinning) | 2.0 | 9.1 | 13.1 | 75.8 |
| 26 | Gambling/poker in casino or video lottery terminal^a^ | 2.0 | 5.1 | 12.1 | 80.8 |
| 27 | Using drugs/alcohol with others^a^ | 9.1 | 20.2 | 23.2 | 47.5 |

^a^ Potential addictive behaviours (Saunders, 2017^1^)

**Supplementary Table 1b. Frequency of prosocial activities in Chennai at Month 6 of follow-up**

| **No.** | **Prosocial activity** | **Frequency (%)** | | | |
| --- | --- | --- | --- | --- | --- |
|  |  | **Often** | **Sometimes** | **Rarely** | **Never** |
| 1 | Going to the cinema/movies | 12.2 | 22.0 | 17.1 | 48.8 |
| 2 | Theatre/concert | 9.8 | 7.3 | 14.6 | 68.3 |
| 3 | Watching an indoor sport in person, not on TV (e.g., chess, table tennis, wrestling) | 5.7 | 9.8 | 19.5 | 65.0 |
| 4 | Watching an outdoor sport in person, not on TV (e.g., football, cricket) | 3.3 | 9.8 | 19.5 | 67.5 |
| 5 | Art gallery/museum | 1.6 | 13.8 | 10.6 | 74.0 |
| 6 | Exhibition | 13.0 | 8.9 | 11.4 | 66.7 |
| 7 | Visiting places of interest (e.g., beach, parks) | 17.1 | 23.6 | 18.7 | 40.7 |
| 8 | Meetings, talks, etc. (outside the house) | 18.7 | 11.4 | 11.4 | 58.5 |
| 9 | Taking a class (outside the house) | 14.6 | 4.9 | 17.1 | 63.4 |
| 10 | Visiting relatives in their homes | 22.0 | 25.2 | 17.1 | 35.8 |
| 11 | Being visited by relatives | 27.6 | 29.3 | 15.4 | 27.6 |
| 12 | Visiting/hanging out with friends (including boyfriends/girlfriends) | 6.5 | 18.7 | 11.4 | 63.4 |
| 13 | Parties (e.g., birthday parties) | 4.9 | 14.6 | 8.1 | 72.4 |
| 14 | Formal occasions (e.g., wedding) | 14.6 | 14.6 | 22.0 | 48.8 |
| 15 | Disco/nightclub | 1.6 | 3.3 | 16.3 | 78.9 |
| 16 | Club/society | 1.6 | 2.4 | 17.1 | 78.9 |
| 17 | Playing an indoor sport | 5.7 | 8.1 | 26.0 | 60.2 |
| 18 | Playing an outdoor sport | 0.8 | 7.3 | 27.6 | 64.2 |
| 19 | Pub/bar | 2.4 | 4.1 | 18.7 | 74.8 |
| 20 | Eating at a restaurant/outside the house | 17.9 | 22.8 | 21.1 | 38.2 |
| 21 | Religious/spiritual activity outside the home | 31.7 | 22.8 | 13.8 | 31.7 |
| 22 | Video chat (e.g., Skype)/online chatting (e.g., MSN) with relatives | 8.1 | 12.2 | 10.6 | 69.1 |
| 23 | Video chat (e.g., Skype)/online chatting (e.g., MSN) with friends | 8.9 | 12.2 | 12.2 | 66.7 |
| 24 | Working out at the gym | 7.3 | 4.9 | 17.1 | 70.7 |
| 25 | Group activity at the gym (e.g., zumba, spinning) | 6.5 | 2.4 | 18.7 | 72.4 |
| 26 | Gambling/video gambling^a^ | 0.8 | 2.4 | 17.9 | 78.9 |
| 27 | Using drugs/alcohol/smoking cigarettes with friends^a^ | 4.1 | 3.3 | 18.7 | 74.0 |

^a^ Potential addictive behaviours (Saunders, 2017^1^)

**Supplementary Table 1c. Frequency of recreation activities in Montreal at Month 6 of follow-up**

| **No.** | **Recreation activity** | **Frequency (%)** | | | |
| --- | --- | --- | --- | --- | --- |
|  |  | **Often** | **Sometimes** | **Rarely** | **Never** |
| 1 | Playing musical instruments | 10.1 | 6.1 | 13.1 | 70.7 |
| 2 | Playing video games/online games^a^ | 23.2 | 24.2 | 15.2 | 37.4 |
| 3 | Sewing, knitting | 0 | 4.0 | 4.0 | 91.9 |
| 4 | Gardening | 2.0 | 5.1 | 10.1 | 82.8 |
| 5 | Reading magazines/books/newspapers | 18.2 | 36.4 | 23.2 | 22.2 |
| 6 | Watching TV/films/online videos | 59.6 | 28.3 | 6.1 | 6.1 |
| 7 | Listening to music | 49.5 | 32.3 | 11.1 | 7.1 |
| 8 | Cooking (as a recreation) | 14.1 | 24.2 | 28.3 | 33.3 |
| 9 | Household/Do It Yourself/renovation projects (e.g., putting up shelves) | 1.0 | 15.2 | 22.2 | 61.6 |
| 10 | Fixing things (car, bike, household, etc.) | 0 | 12.1 | 21.2 | 66.7 |
| 11 | Walking, hiking, jogging | 20.2 | 36.4 | 27.3 | 16.2 |
| 12 | Driving/cycling (as a recreation/hobby) | 13.1 | 10.1 | 11.1 | 65.7 |
| 13 | Swimming | 2.0 | 13.1 | 13.1 | 71.7 |
| 14 | Shopping (as a recreation/hobby) | 6.1 | 26.3 | 30.3 | 37.4 |
| 15 | Artistic activity (painting, crafts etc.) | 7.1 | 15.2 | 15.2 | 62.6 |
| 16 | Hobby (e.g., collecting things) | 9.1 | 13.1 | 19.2 | 58.6 |
| 17 | Using drugs/alcohol alone^a^ | 6.1 | 11.1 | 13.1 | 69.7 |
| 18 | Surfing internet (as a recreation) | 52.5 | 29.3 | 7.1 | 11.1 |
| 19 | Online gambling (e.g., online poker)^a^ | 3.0 | 3.0 | 6.1 | 87.9 |
| 20 | Online course (e.g., language course) | 1.0 | 3.0 | 7.1 | 88.9 |
| 21 | Facebook, Twitter & other social media | 43.4 | 22.2 | 14.1 | 20.2 |
| 22 | Individual sport/physical activity done alone (e.g., working out at home) | 10.1 | 18.2 | 26.3 | 45.5 |

^a^ Potential addictive behaviours (Saunders, 2017^1^)

**Supplementary Table 1d. Frequency of recreation activities in Chennai at Month 6 of follow-up**

| **No.** | **Recreation activity** | **Frequency (%)** | | | |
| --- | --- | --- | --- | --- | --- |
|  |  | **Often** | **Sometimes** | **Rarely** | **Never** |
| 1 | Playing musical instruments | 10.1 | 6.1 | 13.1 | 70.7 |
| 2 | Playing video games/online games^a^ | 23.2 | 24.2 | 15.2 | 37.4 |
| 3 | Sewing, knitting | 0 | 4.0 | 4.0 | 91.9 |
| 4 | Gardening | 2.0 | 5.1 | 10.1 | 82.8 |
| 5 | Reading magazines/books/newspapers | 18.2 | 36.4 | 23.2 | 22.2 |
| 6 | Watching TV/films/online videos | 59.6 | 28.3 | 6.1 | 6.1 |
| 7 | Listening to music | 49.5 | 32.3 | 11.1 | 7.1 |
| 8 | Cooking (as a recreation) | 14.1 | 24.2 | 28.3 | 33.3 |
| 9 | Household/Do It Yourself/renovation projects (e.g., putting up shelves) | 1.0 | 15.2 | 22.2 | 61.6 |
| 10 | Fixing things (car, bike, household, etc.) | 0 | 12.1 | 21.2 | 66.7 |
| 11 | Walking, hiking, jogging | 20.2 | 36.4 | 27.3 | 16.2 |
| 12 | Driving/cycling (as a recreation/hobby) | 13.1 | 10.1 | 11.1 | 65.7 |
| 13 | Swimming | 2.0 | 13.1 | 13.1 | 71.7 |
| 14 | Shopping (as a recreation/hobby) | 6.1 | 26.3 | 30.3 | 37.4 |
| 15 | Artistic activity (painting, crafts etc.) | 7.1 | 15.2 | 15.2 | 62.6 |
| 16 | Hobby (e.g., collecting things) | 9.1 | 13.1 | 19.2 | 58.6 |
| 17 | Using drugs/alcohol alone^a^ | 6.1 | 11.1 | 13.1 | 69.7 |
| 18 | Surfing internet (as a recreation) | 52.5 | 29.3 | 7.1 | 11.1 |
| 19 | Online gambling (e.g., online poker)^a^ | 3.0 | 3.0 | 6.1 | 87.9 |
| 20 | Online course (e.g., language course) | 1.0 | 3.0 | 7.1 | 88.9 |
| 21 | Facebook, Twitter & other social media | 43.4 | 22.2 | 14.1 | 20.2 |
| 22 | Individual sport/physical activity done alone (e.g., working out at home) | 10.1 | 18.2 | 26.3 | 45.5 |

^a^ Potential addictive behaviours (Saunders, 2017^1^)

**Supplementary Table 1e. Frequency of independence-performance activities in Montreal at Month 6 of follow-up**

| **No.** | **Independence-performance activity** | **Frequency (%)** | | | |
| --- | --- | --- | --- | --- | --- |
|  |  | **Often** | **Sometimes** | **Rarely** | **Never** |
| 1 | Buying items from shops | 16.2 | 41.4 | 29.3 | 13.1 |
| 2 | Washing, tidying up (dishes & apartment/house cleaning) | 34.3 | 33.3 | 18.2 | 14.1 |
| 3 | Showering/bathing | 75.8 | 22.2 | 2.0 | 0 |
| 4 | Washing clothes/doing laundry | 34.3 | 33.3 | 15.2 | 17.2 |
| 5 | Looking for jobs | 23.2 | 28.3 | 18.2 | 30.3 |
| 6 | Looking for school opportunities | 10.1 | 28.3 | 21.2 | 40.4 |
| 7 | Shopping for food/groceries | 25.3 | 45.5 | 16.2 | 13.1 |
| 8 | Cooking meals | 23.2 | 36.4 | 23.2 | 17.2 |
| 9 | Leaving the house alone | 41.4 | 32.3 | 17.2 | 9.1 |
| 10 | Uses buses, trains, the metro | 52.5 | 24.2 | 10.1 | 13.1 |
| 11 | Driving a car, bicycle, etc. to get around | 22.2 | 17.2 | 11.1 | 49.5 |
| 12 | Handling money/credit card | 40.4 | 36.4 | 12.1 | 11.1 |
| 13 | Budgeting | 23.2 | 28.3 | 21.2 | 27.3 |
| 14 | Choosing and buying clothes | 9.1 | 40.4 | 28.3 | 22.2 |
| 15 | Taking care of personal appearance | 35.4 | 46.5 | 14.1 | 4.0 |
| 16 | Ensuring payment of bills | 39.4 | 25.3 | 10.1 | 25.3 |

**Supplementary Table 1f. Frequency of independence-performance activities in Chennai at Month 6 of follow-up**

| **No.** | **Independence-performance activity** | **Frequency (%)** | | | |
| --- | --- | --- | --- | --- | --- |
|  |  | **Often** | **Sometimes** | **Rarely** | **Never** |
| 1 | Buying items from shops | 32.0 | 24.6 | 22.1 | 21.3 |
| 2 | Washing, tidying up (dishes & apartment/house cleaning) | 36.9 | 16.4 | 22.1 | 24.6 |
| 3 | Showering/bathing | 75.6 | 17.1 | 1.6 | 5.7 |
| 4 | Washing clothes/doing laundry | 44.7 | 20.3 | 8.9 | 26.0 |
| 5 | Looking for jobs | 24.4 | 15.4 | 9.8 | 50.4 |
| 6 | Looking for school opportunities | 14.6 | 7.3 | 8.9 | 69.1 |
| 7 | Shopping for food/groceries | 24.4 | 28.5 | 17.1 | 30.1 |
| 8 | Cooking meals | 34.1 | 12.2 | 13.0 | 40.7 |
| 9 | Leaving the house alone | 43.1 | 27.6 | 11.4 | 17.9 |
| 10 | Uses buses, trains, the metro, auto rickshaws | 40.2 | 20.5 | 13.9 | 25.4 |
| 11 | Driving a car, bicycle, etc. to get around | 27.6 | 9.8 | 15.4 | 47.2 |
| 12 | Handling money/credit card | 35.0 | 20.3 | 17.1 | 27.6 |
| 13 | Budgeting | 21.1 | 28.5 | 13.8 | 36.6 |
| 14 | Choosing and buying clothes | 35.8 | 22.8 | 16.3 | 25.2 |
| 15 | Taking care of personal appearance | 47.2 | 30.1 | 8.1 | 14.6 |
| 16 | Ensuring payment of bills | 28.5 | 8.9 | 21.1 | 41.5 |
